# Stranger Than Fiction

**Published:** 1892-07

**Authors:** 


### STRANGER THAN FICTION.

The following is an incident related and vouched for by the Rev. Minot J. Savage, in a recent article published at length in "The Arena:"

"The lady who had this (the following) experience," says Mr. Savage, "was a schoolmate of my brother, and her character and veracity are beyond question."

"In June, 1886, she was a patient in the family of a physician in a well-known city in a neighboring state. She was suffering much from mental depression, feeling assured in her own mind that she had an ovarian tumor. On this particular day, she was lying alone in her room, unusually oppressed by foreboding fears. Lying thus, absorbed in thoughts of her own condition, she suddenly became conscious as of an open map of the United States being spread before her. Her attention was particularly directed to Virginia, and then westward to, as she then thought, Ohio. At the same time she heard the name "McDowell." At once she thought of General McDowell, as the only one she knew of by that name. But a calm, gentle voice seemed to reply to her unspoken thought, "No, I am not General McDowell, but a physician. I was the first advocate and practitioner of ovarian surgery. By the urgent request of your friends, I have examined your case very carefully. Rest assured madam; your malady is not of that character. In time you will regain your health, but never be very strong.

With a feeling of awe, gratitude, and wonder which, she says she could not attempt to express, she rose from the couch on which she was lying, and went at once to the doctor's office in another part of the house. At once she related what had occurred, and asked, "Am I right?" The physician, a lady, went to her library and took down her Medical Encyclopaedia. From this she read, 'Ephraim McDowell, born in Virginia, settled in Kentucky. He performed the first operation in ovarian surgery that is recorded in this country."

"She was correct, therefore, in every particular, except the substituting Ohio for Kentucky, and this is quite natural as it is the next adjoining state."

I have been in recent correspondence with the physician in whose house she was at the time. This physician completely confirms all the facts, and testifies in the most emphatic way to the noble character and unquestioned veracity of her patient. And yet, though she offers no other theory, she is strongly opposed to any explanation that calls for the agency of any supernormal intelligence. This, however, grows out of the fact that she has always been bitterly prejudiced against everything of the kind.

And lastly, both the physician and her patient are perfectly assured that the name of Dr. McDowell and his work as a surgeon were entirely unknown to the teller of this experience at the time when the voice was heard."

A case similar to the foregoing is vouched for by a relative of the writer. Some years ago, an only child of parents temporarily residing in the little village of Glen Coe, Canada, was taken suddenly and violently ill and great fears were entertained lest the attack should terminate fatally. The nearest points to obtain satisfactory medical aid were Windsor on the one hand or London on the other, and the parents were sitting by the little sufferer in solemn consultation as to the best thing to be done, when with a sudden impulse and strange in manner and voice, the mother spoke substantially as follows: "You need not go to a distance for help, I will take charge of this case." Here a simple remedy was prescribed easily obtainable at home. On being questioned as to the identity of the mysterious presence, the answer promptly came "I am Sir Vincent Manderville, formerly physician in chief to his majesty, King George the 3d. I sought the best means at home and abroad to gain a knowledge of my profession, and for more than a year walked the hospitals of Paris. Follow my prescription and your child will surely recover."

It is needless to say that the direction given was sacredly observed; the child, in the person of an accomplished young lady, is living to-day, and Doctor Manderville is still an unquestioned authority upon all medical points in the household.

